# 5,12-Diselena-3,4,13,14-tetra­aza­tri­cyclo[9.3.0.0^2,6^]tetra­deca-3,13-diene

**DOI:** 10.1107/S2414314620015850

**Published:** 2020-12-11

**Authors:** Heiner Detert, Dieter Schollmeyer

**Affiliations:** a Johannes Gutenberg University Mainz, Department of Chemistry, Duesbergweg 10-14, 55099 Mainz, Germany; Goethe-Universität Frankfurt, Germany

**Keywords:** crystal structure, heterocycles, medium-sized ring, selenium

## Abstract

The title compound, C_8_H_8_N_4_Se_2_, crystallizes in a non-symmetrical conformation with a dihedral angle between the heterocycles of 45.0 (3)° and a nearly strain-free tetra­methyl­ene tether. The crystal studied was non-merohedrally twinned with a fractional contribution of 0.342 (3) for the minor twin component.

## Abstract 2

The title compound crystallizes in a non-symmetrical conformation with a dihedral angle between the heterocycles of 45.0 (3)° and a nearly strain-free tetra­methyl­ene tether.

## Graphical abstract

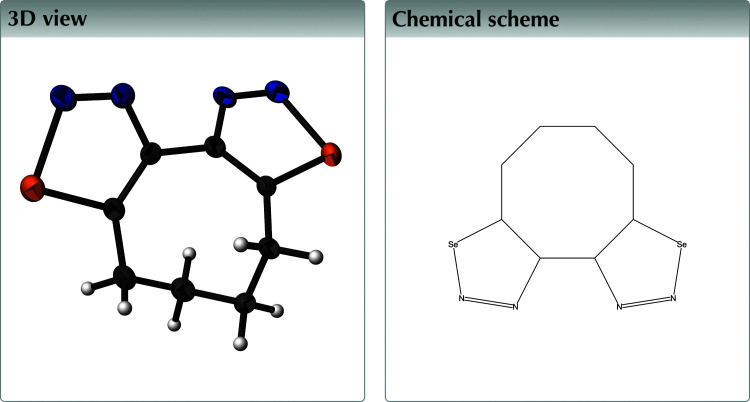

## Structure description

1,2,3-Selena­diazo­les are synthesized from SeO_2_-oxdidation of semicarbazones (Yalpani *et al.*, 1971 ▸; Al-Smadi & Ratrout, 2004 ▸) and are important inter­mediates for the synthesis of medium-sized (Meier, 1972 ▸) heterocyclic (Detert, 2011 ▸) and strained cyclo­alkynes (Bissinger *et al.*, 1988 ▸). Bis-selena­diazo­les have been used as inter­mediates for the synthesis of medium-sized cyclo­alkadiynes (Gleiter *et al.*, 1988 ▸).

**Figure 1:**
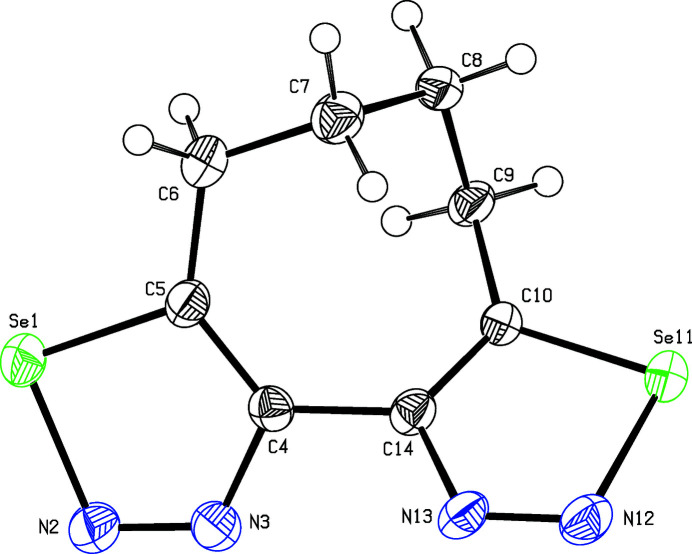
Perspective view of the title compound. Displacement ellipsoids are drawn at the 50% probability level.

**Figure 2:**
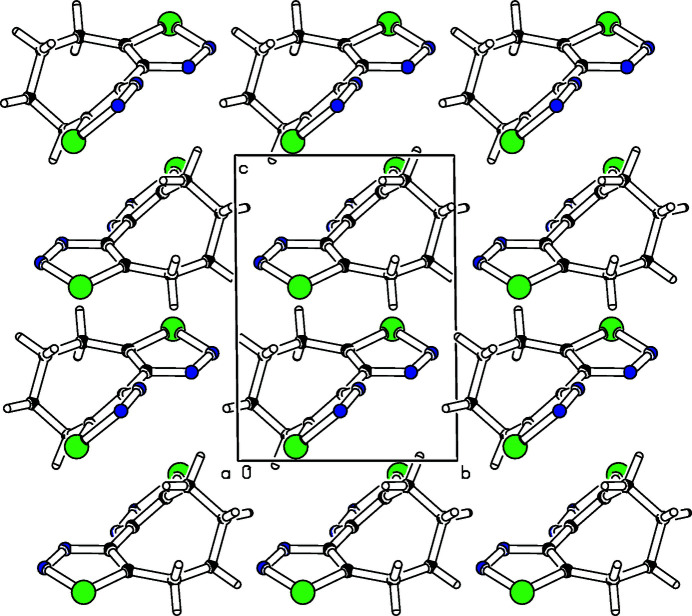
Partial packing diagram of the title compound. View is along the *b* axis.

The selena­diazole rings in the title compound (Fig. 1 ▸) are essentially planar and include a torsion angle of N13—C14—C4—N3 = −43.6 (10)°. This torsion angle is significantly smaller than the corresponding torsion angle (58.2°) in a dibenzo­cyclo­octa-1,3-diene (Janhsen *et al.*, 2017 ▸). In the tetra­methyl­ene tether, the dihedral angle at C8—C9—C10—C14 [84.9 (10)°] shows the largest deviation from the ideal value of 60° whereas C6—C7—C8—C9 matches this value nearly perfectly: −59.7 (11)°. Contrary to the formal symmetry, the conformer in the crystal shows neither a *C*_2_ axis nor a mirror plane. Two mol­ecules of the title compound fill the unit cell, and these are related by a center of inversion. One hydrogen atom at C7 points to the center of a selena­diazole of the neighbouring mol­ecule, thus keeping the rings at a distance (Fig. 2 ▸).

## Synthesis and crystallization

The title compound was prepared from cyclo­octa­none *via* oxidation with selenium dioxide to suberil, the formation of bis-semicarbazone and oxidation/cyclization with selenic acid. 5.1 g of bis-semicarbazone in 100 ml of 1,4-dioxane were stirred for 7 d after the addition of 6.6 g of SeO_2_ in 10 ml of water. The mixture was concentrated to 60 ml, diluted with water (100 ml) and extracted with chloro­form (2×). The pooled solutions were dried, concentrated and the residue purified *via* chromatography (SiO_2_, toluene/ethyl acetate 10/1, *R*_f_ = 0.35). Recrystallization from the mixed solvents of chloro­form/propanol-2 yielded colorless crystals with m.p. = 453 K (under explosion). ^13^C NMR data are consistent with data given by Meier (Meier *et al.*, 1981 ▸)

^1^H NMR (CDCl_3_, 400 MHz) 3.15 (*broad s*, 4 H, H_2_C-7, 10); 1.90 (*broad s*), 4 H, H_2_C-8,9); ^13^C NMR: 164.1, 151.9 (C-1,2,6,11), 26.8 (C-7, 10); 25.9 (C-8,9); IR (KBr): 2960, 1480, 1450, 1345, 1304, 1266, 844; ^77^Se NMR (CDCl_3_. 73 MHz, SeO_2_/D_2_O as reference): 238.9; ^15^N NMR (CDCl_3_, CH_3_NO_2_ as reference, 40.3 MHz): 87.1, 83.3; UV–vis (EtOH): 212 nm (4.,38), 243 (4.04), 297 (3.53); MS: *m*/*z* = 264 (17%, Se_2_ pattern), 236 (17%, Se_2_ pattern); 118 (21%, Se pattern), 104 (81%, C_8_H_8_); 103 (100%).

## Refinement

Crystal data, data collection and structure refinement details are summarized in Table 1 ▸. The crystal studied was non-merohedrally twinned with a fractional contribution of 0.342 (3) for the minor twin component.

## Supplementary Material

Crystal structure: contains datablock(s) I, global. DOI: 10.1107/S2414314620015850/bt4104sup1.cif

Structure factors: contains datablock(s) I. DOI: 10.1107/S2414314620015850/bt4104Isup2.hkl

Supporting information file. DOI: 10.1107/S2414314620015850/bt4104Isup3.cml

CCDC reference: 2048056

Additional supporting information:  crystallographic information; 3D view; checkCIF report

## Figures and Tables

**Table 1:** Experimental details

Crystal data	
Chemical formula	C_8_H_8_N_4_Se_2_
*M*_r_	318.10
Crystal system, space group	Triclinic, *P* 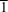
Temperature (K)	120
*a*, *b*, *c* (Å)	7.5350 (18), 7.6723 (17), 9.372 (2)
α, β, γ (°)	90.136 (18), 90.773 (19), 118.555 (17)
*V* (Å^3^)	475.8 (2)
*Z*	2
Radiation type	Mo *K*α
μ (mm^−1^)	7.73
Crystal size (mm)	0.45 × 0.23 × 0.22

Data collection	
Diffractometer	Stoe IPDS 2T
Absorption correction	Integration
*T*_min_, *T*_max_	0.093, 0.252
No. of measured, independent and observed [*I* > 2σ(*I*)] reflections	6775, 6775, 5946
*R*_int_	0.046
(sin θ/λ)_max_ (Å^−1^)	0.666

Refinement	
*R*[*F* ^2^ > 2σ(*F* ^2^)], *wR*(*F* ^2^), *S*	0.065, 0.217, 1.12
No. of reflections	6775
No. of parameters	128
H-atom treatment	H-atom parameters constrained
Δρ_max_, Δρ_min_ (e Å^−3^)	1.68, −1.50

Computer programs: *X-AREA* *WinXpose*, *Recipe* and *X-AREA* *Integrate* (Stoe & Cie, 2019 ▸), *SIR2004* (Burla *et al.*, 2005 ▸), *SHELXL2018/3* (Sheldrick, 2015 ▸) and *PLATON* (Spek, 2020 ▸).

## full crystallographic data

### Crystal data

**Table:**

C_8_H_8_N_4_Se_2_	*Z* = 2
*M**_r_* = 318.10	*F*(000) = 304
Triclinic, *P*1	*D*_x_ = 2.220 Mg m^−^^3^
*a* = 7.5350 (18) Å	Mo *K*α radiation, λ = 0.71073 Å
*b* = 7.6723 (17) Å	Cell parameters from 5222 reflections
*c* = 9.372 (2) Å	θ = 3.0–28.6°
α = 90.136 (18)°	µ = 7.73 mm^−^^1^
β = 90.773 (19)°	*T* = 120 K
γ = 118.555 (17)°	Needle, brown
*V* = 475.8 (2) Å^3^	0.45 × 0.23 × 0.22 mm

### Data collection

**Table:**

Stoe IPDS 2T diffractometer	6775 independent reflections
Radiation source: sealed X-ray tube, 12 x 0.4 mm long-fine focus	5946 reflections with *I* > 2σ(*I*)
Detector resolution: 6.67 pixels mm^-1^	*R*_int_ = 0.046
rotation method scans	θ_max_ = 28.3°, θ_min_ = 3.0°
Absorption correction: integration	*h* = −9→9
*T*_min_ = 0.093, *T*_max_ = 0.252	*k* = −10→10
6775 measured reflections	*l* = −12→12

### Refinement

**Table:**

Refinement on *F*^2^	Primary atom site location: structure-invariant direct methods
Least-squares matrix: full	Hydrogen site location: inferred from neighbouring sites
*R*[*F*^2^ > 2σ(*F*^2^)] = 0.065	H-atom parameters constrained
*wR*(*F*^2^) = 0.217	*w* = 1/[σ^2^(*F*_o_^2^) + (0.1209*P*)^2^ + 3.1505*P*] where *P* = (*F*_o_^2^ + 2*F*_c_^2^)/3
*S* = 1.12	(Δ/σ)_max_ < 0.001
6775 reflections	Δρ_max_ = 1.68 e Å^−^^3^
128 parameters	Δρ_min_ = −1.50 e Å^−^^3^
0 restraints	

### Special details

**Table:**

Geometry. All esds (except the esd in the dihedral angle between two l.s. planes) are estimated using the full covariance matrix. The cell esds are taken into account individually in the estimation of esds in distances, angles and torsion angles; correlations between esds in cell parameters are only used when they are defined by crystal symmetry. An approximate (isotropic) treatment of cell esds is used for estimating esds involving l.s. planes.
Refinement. Refined as a 2-component twin.Hydrogen atoms attached to carbons were placed at calculated positions and were refined in the riding-model approximation with C–H = 0.95 Å, and with *U*_iso_(H) = 1.2 *U*_eq_(C).

### Fractional atomic coordinates and isotropic or equivalent isotropic displacement parameters (Å^2^)

**Table:**

	*x*	*y*	*z*	*U*_iso_*/*U*_eq_	
Se1	0.71557 (14)	0.29021 (14)	0.56893 (9)	0.0299 (3)	
N2	0.4663 (14)	0.1129 (13)	0.6483 (9)	0.0330 (17)	
N3	0.3899 (14)	0.2082 (12)	0.7102 (8)	0.0300 (16)	
C4	0.4967 (13)	0.4132 (13)	0.7084 (8)	0.0219 (15)	
C5	0.6788 (13)	0.4941 (14)	0.6393 (8)	0.0245 (16)	
C6	0.8389 (16)	0.7040 (16)	0.6103 (10)	0.0323 (19)	
H6A	0.864929	0.714893	0.506599	0.039*	
H6B	0.965074	0.725639	0.659402	0.039*	
C7	0.7975 (16)	0.8732 (15)	0.6540 (9)	0.0309 (18)	
H7A	0.653666	0.832240	0.633311	0.037*	
H7B	0.879991	0.989880	0.594356	0.037*	
C8	0.8430 (14)	0.9360 (14)	0.8119 (9)	0.0262 (16)	
H8A	0.988852	0.985256	0.831092	0.031*	
H8B	0.814767	1.047758	0.828919	0.031*	
C9	0.7226 (14)	0.7718 (14)	0.9176 (8)	0.0257 (16)	
H9A	0.772439	0.673575	0.917303	0.031*	
H9B	0.743231	0.829398	1.014935	0.031*	
C10	0.5016 (13)	0.6699 (13)	0.8799 (8)	0.0222 (15)	
Se11	0.31509 (15)	0.73496 (15)	0.95420 (9)	0.0304 (3)	
N12	0.1178 (13)	0.5308 (14)	0.8370 (9)	0.0327 (16)	
N13	0.1976 (12)	0.4468 (13)	0.7656 (8)	0.0277 (15)	
C14	0.4026 (13)	0.5159 (14)	0.7841 (8)	0.0233 (16)	

### Atomic displacement parameters (Å^2^)

**Table:**

	*U*^11^	*U*^22^	*U*^33^	*U*^12^	*U*^13^	*U*^23^
Se1	0.0262 (5)	0.0322 (5)	0.0348 (5)	0.0169 (4)	0.0023 (4)	−0.0034 (4)
N2	0.034 (5)	0.028 (4)	0.035 (4)	0.013 (4)	0.011 (3)	0.004 (3)
N3	0.033 (4)	0.026 (4)	0.027 (3)	0.011 (4)	0.005 (3)	0.000 (3)
C4	0.023 (4)	0.023 (4)	0.019 (3)	0.010 (3)	−0.002 (3)	−0.002 (3)
C5	0.021 (4)	0.029 (4)	0.023 (3)	0.012 (4)	0.001 (3)	−0.002 (3)
C6	0.030 (5)	0.033 (5)	0.032 (4)	0.014 (4)	0.009 (4)	−0.001 (4)
C7	0.031 (5)	0.032 (5)	0.028 (4)	0.014 (4)	0.005 (3)	0.004 (3)
C8	0.023 (4)	0.025 (4)	0.026 (3)	0.007 (4)	0.002 (3)	−0.004 (3)
C9	0.020 (4)	0.031 (4)	0.020 (3)	0.008 (4)	0.003 (3)	−0.002 (3)
C10	0.022 (4)	0.022 (4)	0.021 (3)	0.009 (3)	0.004 (3)	−0.001 (3)
Se11	0.0275 (5)	0.0315 (5)	0.0321 (5)	0.0138 (4)	0.0066 (4)	−0.0050 (4)
N12	0.021 (4)	0.038 (5)	0.036 (4)	0.012 (4)	0.002 (3)	−0.003 (3)
N13	0.019 (4)	0.034 (4)	0.027 (3)	0.011 (3)	0.003 (3)	0.001 (3)
C14	0.021 (4)	0.028 (4)	0.020 (3)	0.011 (4)	0.001 (3)	0.000 (3)

### Geometric parameters (Å, º)

**Table:**

Se1—C5	1.837 (9)	C7—H7B	0.9900
Se1—N2	1.879 (9)	C8—C9	1.526 (12)
N2—N3	1.269 (11)	C8—H8A	0.9900
N3—C4	1.383 (12)	C8—H8B	0.9900
C4—C5	1.378 (13)	C9—C10	1.499 (12)
C4—C14	1.473 (11)	C9—H9A	0.9900
C5—C6	1.509 (14)	C9—H9B	0.9900
C6—C7	1.529 (13)	C10—C14	1.376 (12)
C6—H6A	0.9900	C10—Se11	1.845 (8)
C6—H6B	0.9900	Se11—N12	1.895 (9)
C7—C8	1.538 (12)	N12—N13	1.267 (11)
C7—H7A	0.9900	N13—C14	1.381 (11)
			
C5—Se1—N2	87.9 (4)	C9—C8—C7	114.7 (8)
N3—N2—Se1	110.2 (7)	C9—C8—H8A	108.6
N2—N3—C4	117.8 (8)	C7—C8—H8A	108.6
C5—C4—N3	115.8 (8)	C9—C8—H8B	108.6
C5—C4—C14	128.7 (8)	C7—C8—H8B	108.6
N3—C4—C14	115.5 (8)	H8A—C8—H8B	107.6
C4—C5—C6	133.7 (8)	C10—C9—C8	111.1 (6)
C4—C5—Se1	108.3 (7)	C10—C9—H9A	109.4
C6—C5—Se1	118.0 (6)	C8—C9—H9A	109.4
C5—C6—C7	118.1 (8)	C10—C9—H9B	109.4
C5—C6—H6A	107.8	C8—C9—H9B	109.4
C7—C6—H6A	107.8	H9A—C9—H9B	108.0
C5—C6—H6B	107.8	C14—C10—C9	126.9 (8)
C7—C6—H6B	107.8	C14—C10—Se11	108.1 (6)
H6A—C6—H6B	107.1	C9—C10—Se11	125.0 (6)
C6—C7—C8	114.7 (7)	C10—Se11—N12	87.4 (4)
C6—C7—H7A	108.6	N13—N12—Se11	110.3 (6)
C8—C7—H7A	108.6	N12—N13—C14	117.5 (8)
C6—C7—H7B	108.6	C10—C14—N13	116.6 (8)
C8—C7—H7B	108.6	C10—C14—C4	124.8 (8)
H7A—C7—H7B	107.6	N13—C14—C4	118.5 (8)
			
C5—Se1—N2—N3	0.2 (6)	C8—C9—C10—Se11	−94.1 (8)
Se1—N2—N3—C4	0.1 (9)	C14—C10—Se11—N12	0.0 (6)
N2—N3—C4—C5	−0.5 (11)	C9—C10—Se11—N12	179.1 (7)
N2—N3—C4—C14	−178.9 (7)	C10—Se11—N12—N13	0.2 (6)
N3—C4—C5—C6	179.1 (8)	Se11—N12—N13—C14	−0.4 (10)
C14—C4—C5—C6	−2.7 (14)	C9—C10—C14—N13	−179.3 (7)
N3—C4—C5—Se1	0.6 (8)	Se11—C10—C14—N13	−0.2 (9)
C14—C4—C5—Se1	178.8 (6)	C9—C10—C14—C4	4.8 (13)
N2—Se1—C5—C4	−0.4 (6)	Se11—C10—C14—C4	−176.1 (6)
N2—Se1—C5—C6	−179.2 (7)	N12—N13—C14—C10	0.4 (11)
C4—C5—C6—C7	−5.1 (14)	N12—N13—C14—C4	176.6 (7)
Se1—C5—C6—C7	173.2 (6)	C5—C4—C14—C10	−46.0 (12)
C5—C6—C7—C8	82.6 (11)	N3—C4—C14—C10	132.3 (9)
C6—C7—C8—C9	−59.7 (11)	C5—C4—C14—N13	138.2 (8)
C7—C8—C9—C10	−49.6 (10)	N3—C4—C14—N13	−43.6 (10)
C8—C9—C10—C14	84.9 (10)		
